# An integrated physiology, cytology, and proteomics analysis reveals a network of sugarcane protoplast responses to enzymolysis

**DOI:** 10.3389/fpls.2022.1066073

**Published:** 2022-11-28

**Authors:** Demei Zhang, Rui Wang, Jiming Xiao, Shuifang Zhu, Xinzhu Li, Shijian Han, Zhigang Li, Yang Zhao, M. J. I. Shohag, Zhenli He, Suli Li

**Affiliations:** ^1^ Guangxi Key Laboratory of Sugarcane Biology, College of Agriculture, Guangxi University, Nanning, China; ^2^ Key Laboratory of Crop Cultivation and Tillage, College of Agriculture, Guangxi University, Nanning, China; ^3^ School of Biomedical Engineering, South-Central Minzu University, Wuhan, China; ^4^ Institute of Food and Agricultural Sciences (IFAS) Indian River Research and Education Center, University of Florida, Fort Pierce, FL, United States

**Keywords:** protoplast, enzymolysis, sugarcane, proteomics, cytology, oxidative stress

## Abstract

The protoplast experimental system eis an effective tool for functional genomics and cell fusion breeding. However, the physiological and molecular mechanisms of protoplast response to enzymolysis are not clear, which has become a major obstacle to protoplast regeneration. Here, we used physiological, cytological, proteomics and gene expression analysis to compare the young leaves of sugarcane and enzymolized protoplasts. After enzymatic digestion, we obtained protoplasts with viability of > 90%. Meanwhile, the content of malondialdehyde, an oxidation product, increased in the protoplasts following enzymolysis, and the activity of antioxidant enzymes, such as peroxidase (POD), catalase (CAT), acid peroxidase (APX), and O^2-^, significantly decreased. Cytologic analysis results showed that, post enzymolysis, the cell membranes were perforated to different degrees, the nuclear activity was weakened, the nucleolus structure was not obvious, and the microtubules depolymerized and formed several short rod-like structures in protoplasts. In this study, a proteomics approaches was used to identify proteins of protoplasts in response to the enzymatic digestion process. GO, KEGG, and KOG enrichment analyses revealed that the abundant proteins were mainly involved in bioenergetic metabolism, cellular processes, osmotic stress, and redox homeostasis of protoplasts, which allow for protein biosynthesis or degradation. RT-qPCR analysis revealed that the expression of osmotic stress resistance genes, such as *DREB, WRKY, MAPK4*, and *NAC*, was upregulated, while that of key regeneration genes, such as *CyclinD3, CyclinA, CyclinB*, *Cdc2, PSK, CESA*, and *GAUT*, was significantly downregulated in the protoplasts. Hierarchical clustering and identification of redox proteins and oxidation products showed that these proteins were involved in dynamic networks in response to oxidative stress after enzymolysis. Our findings can facilitate the development of a standard system to produce regenerated protoplasts using molecular markers and antibody detection of enzymolysis.

## 1 Introduction

Protoplasts, somatic plant cells from which the cell wall has been enzymatically or physically removed (Chupeau et al., 2013), can maintain the physiological and cellular processes of whole plants (Xu et al., 2021). As a result, the protoplast experimental system has become a strong functional genomics tool for investigating protein-protein interactions, protein localization, and signaling pathways involved in plant physiology, innate immunity, growth, and development (Page et al., 2019). In addition, somatic hybridization supplies a practical tool in the breeding of different crop species (Johnson and Veilleux, 2010) and circumvents the prezygotic or postzygotic barriers associated with sexual hybridization. It can create different homokaryon or heterokaryon types, as well as alloplasmic hybrids (cybrids) (Xia, 2009). Somatic hybridization breeding has been reported for various plant species, including rice (Ma et al., 2020) and maize (Zhou et al., 2008). However, regeneration presents a typical bottleneck in somatic hybridization breeding programs, forcing researchers to adopt novel approaches, such as identifying the optimal donor material, source, protoplast isolation method, and culture method (Davey et al., 2005).

Enzymolysis is the first step in somatic hybridization breeding, and the degree of protoplast damage is key in determining the regeneration of protoplasts (Chupeau et al., 2013). To obtain high regeneration rates, it was recommended that young and non-stressed tissues should be used for protoplast isolation (Chupeau et al., 2013) and that plant growth conditions should be adjusted to avoid premature cell death (Horii and Marubashi, 2005). However, plant protoplasts remain intact only at a proper osmotic pressure during enzymolysis, and the osmotic pressure must be adjusted to obtain the maximum viable protoplast (Li et al., 2020). The cell wall is degraded under osmotic pressure usually *via* a sugar such as mannitol, sucrose, or sorbitol (Estavillo et al., 2014). This prevents protoplast damage caused by osmolality variations between the cell interior and the digesting medium (Estavillo et al., 2014). The suggested link between osmolarity decrease or increase and chromatin over condensation may explain the intricate interaction between genetic background and environmental factors during early protoplasts regeneration (Chupeau et al., 2013).

When attempting to isolate the cytosolic fraction of plant cells, it is critical to start with a pure sample of intact protoplasts, as many break during digestion. The damage produced during the isolation of protoplasts can have a stressful effect on cells during the subsequent culture, and triggers dedifferentiation (Zhang et al., 2022). The oxidative stress evoked during protoplast isolation and their culture may contribute to the recalcitrance of protoplasts (Petřivalský et al., 2012). It has been reported that reactive oxygen species (ROS) produced by cells treated with xylanase or pectin lyase can damage rice protoplasts (Ishii, 1988), and that adding superoxide dismutase (SOD) and catalase (CAT) to the rice protoplast isolation medium improve protoplast viability (Pasternak et al., 2005). Different levels of ROS, antioxidant enzymes and scavengers influence the regeneration capability of protoplasts derived from the cells of different species (Petřivalský et al., 2012). Accumulated ROS are associated with increased lipid peroxidation (Papadakis et al., 2001) and initiat apoptosis-like cell death in cultured protoplasts (Yasuda et al., 2007).

The removal of the cell wall imposes a tremendous challenge to cells, hence plant cells respond to cell wall removal at every level of the regulatory hierarchy in the nucleus (Mujahid et al., 2013). As a result, the enzymatic solution disrupts not only the physiologic function but also the cytologic at molecular mechanisms of protoplasts (Fu and Zhuang, 2001). Isolation generates structural variability, thereby causing anomalies during cell division (Tylicki et al., 2002), as well as in plants regenerated from protoplasts (Cambecedes et al., 1988). These anomalies often hinder the introduction of new plant varieties obtained *via in vitro* protoplast fusion (Handley et al., 1986). Morphological observation showed cortical microtubules in the protoplasts at all times, following examination of osmotically ruptured protoplasts (Marchant and Hines, 1979) The early devel opmental stages of protoplasts are accompanied by large-scale chromatin remodeling (Zhang et al., 2022) and major transcriptional changes (Hayat et al., 2022). Sharma et al. (2011) examined transcriptomic responses to enzymatic removal of the cell wall and found that among differentially regulated genes, the kinases, transcription factors, and genes predicted to be involved in cell wall-related functions were enriched (Mujahid et al., 2013). Differential expression of proteomes, including transcription factors, histones, histone domain-containing proteins, and histone modification enzymes, occurred in the nucleus in response to the removal of the cell wall in rice suspension cells (Mujahid et al., 2013). Gene ontology analysis of the differentially expressed proteins indicates that chromatin and nucleosome assembly, protein-DNA complex assembly, and DNA packaging are tightly associated with cell wall removal (Mujahid et al., 2013). Moreover, osmotic stress due to protoplast separation altered the expression of several resistance genes, leading to browning (Ondřej et al., 2009). However, data on the mechanisms by which plant cells sense enzymatic removal of the cell wall and transduce corresponding signals to regulate cellular responses to maintain protoplast integrity are limited. Advances in molecular biology techniques can increase our understanding of plant cryobiology. By combining physiologic, cytologic, and molecular biologic approaches, an array of methods are available to elucidate how cells are protected during the enzymolysis process.

Sugarcane (*Saccharum* spp.) is an industrially important major sugar crop in tropical and subtropical areas, contributing 70% of global sugar production (Pang et al., 2021). It is also utilized in the production of biofuel, ethanol, and other products such as paper, plywood, animal feed, and industrial enzymes (Sheen, 2001). Somatic hybridization in sugarcane allows for broadening germplasm base. Current research on sugarcane protoplasts has primarily focused on optimizing the enzymolysis conditions, such as the enzymolysis method, medium, hormone, and protoplast concentration. The highly viable sugarcane protoplasts obtained *via* enzymolysis of sugarcane young leaves using the optimal mannitol concentration showed severe browning at a later stage, and cells were unable to divide continuously, which greatly hindered the regeneration of sugarcane protoplasts (Meena et al., 2022). If late protoplast browning is not addressed, the regeneration of sugarcane protoplasts will be a significant problem (Meena et al., 2022). Sugarcane protoplasts are inevitably affected by external conditions during enzymolysis, which affects the expression of relevant genes and the regeneration ability of the protoplasts (Tessadori et al., 2007).

Previously, we have conducted transcriptome sequencing on sugarcane young leaves and protoplasts after enzymolysis, and observed significant differences in the differentially expressed genes (DEGs) and the DEGs categories (Zhang et al., 2022). However, the molecular mechanism by which protein regulatory networks regulate cell division, differentiation, and further rooting into seedlings in sugarcane protoplasts remains unknown. Integrated cytologic, physiologic, and proteomic analyses of the responses of sugarcane young leaves to enzymolysis will provide key information regarding the mechanisms of recovery pathways in protoplast systems that succeed or fail to survive enzymolysis. In this study, we used physiologic, cytologic, proteomic, and PCR analyses to examine the differences in sugarcane protoplasts before and after enzymolysis. We performed functional enrichment analysis of differential genes and functional annotation of proteins to elucidate the molecular, physiologic, and cytologic mechanisms hindering the regeneration of sugarcane protoplasts. This study provides the relevant criteria and theoretical foundation for constructing a standard system for producing regenerated protoplasts using molecular markers and antibody detection of enzymolysis.

## 2 Materials and methods

### 2.1 Plant material, protoplast material acquisition

The sugarcane variety ROC22 planted in the sugarcane (*Saccharum officinarum*) experimental base of the College of Agriculture, Guangxi University, Guangxi, PR. China. The young leaves were sampled at the early elongation stage. Collected samples were quickly frozen in liquid nitrogen and stored at -80°C freezer for further use.

Protoplasts were isolated from sugarcane young leaves as previously described (Zhang et al., 2022). Briefly, the robust tail sheaths of sugarcane at the initial elongation stage wereselected as materials for enzymolysis. At first, outer two to three layers of the leaf sheaths were peeled off and then sterilized with 75% alcohol for 30 s. The outer layer and leaf sheaths at both ends were removed after three washes with sterile water to reveal the light-yellow central leaves. The young leaves 1−5 cm above the growing point were cut into thin slices with a thickness of approximately 1 mm. Next, 0.5 g of young leaves were collected and added to 5 mL of CPW solution (containing 13% mannitol, pH 5.8). After plasmolysis for 0.5–1 h, the CPW (containing 13% mannitol) solution was removed, and 5 mL of enzymolysis solution was added to allow enzymolysis at 28°C for 4 h. With 0.5M of mannitol in the digestion buffer, highest protoplast yield and viability was further observed using an enzyme composition containing 3% (m/v) cellulase R-10 and 1.7% (m/v) pectinase R-10. The protoplast suspension was then filtered through 100- and 200-mesh cell sieves subsequently, and the protoplasts were purified using gradient centrifugation (750 rpm/min, 5 min).

### 2.2 Protoplast viability testing

Protoplast viability was determined using fluorescein diacetate (FDA): 100 uL of protoplast suspension was aspirated in a 2 mL centrifuge tube, 2 uL of 5 mg/mL FDA was added, mixed, and incubated at room temperature for 5 min. Green protoplasts were observed and photographed under blue excitation light using a confocal laser microscope (Nikon, Beijing, China). The green- fluorescent active protoplasts were observed and photographed under blue excitation light using a confocal laser microscope (Nikon, Beijing, China). The FDA stock solution was dissolved in acetone to 5 g/L and stored at -20°C. The FDA working solution was made by diluting the above stock solution with phosphate buffer solution (PBS) to 5 mg/mL. The total number of cells in the samples measured in this study was 2.5 × 10^6^ g/FW.

### 2.3 Characterization *via* scanning electron microscopy

The enzymatically digested protoplasts were put into 2.5% glutaraldehyde fixative (pH 7.2) at 4°C and fixed for more than 2 h; washed 3 times with 0.1 mol/L PBS (pH 7.2) for 10 min each; fixed for 3 h after 1% starvation acid; washed again 3 times with PBS for 10 min each; graded with 30%, 50%, 70%, 90% and 100% alcohol After dehydration, the samples were dried in a Leica (EM CPD300, Weztlar, GER) automatic critical point drier. The dried samples were fixed on the sample tray with conductive double-sided adhesive and dried under vacuum (1 × 10 ^-6^ kPa) for 0.5 h to increase the adhesion of the sample to the conductive adhesive. The sample is gently held with forceps and pulled upward, and the pulled off tissue is partly adhered to the conductive adhesive and the other part is made to adhere to the conductive adhesive under the dissecting microscope with the fracture side up. After the samples were pasted, the samples were sprayed with gold in a Leica (EM ACE600, Weztlar, GER) ion sputter coater with a coating thickness of 22 nm, and the samples were observed and photographed using a Hitachi (S-3400N, Tokyo, JPN) scanning electron microscope (Lu et al., 2021).

### 2.4 Nucleus morphology of sugarcane young leaves and protoplasts

Chromosomes were examined in a dark room. First, 30 µL of DAPI (0.5 µg/mL) drops per sheet were applied to the microscopically examined chromosome films, covered with coverslips, and observed using a fluorescence microscope (Leica DMRA2 Microscope, Weztlar,GER) and the images were captured using the SH DC 350F camera system (Leica QFI, Weztlar, GER). The average pixel values of chromosomes were measured using Leica (CW4000, Weztlar, GER).

### 2.5 Changes in the microtubule skeleton of sugarcane young leaves and protoplasts

The frozen sectioning method combined with indirect immunofluorescence technique and DAPI staining was used to observe the arrangement of the cellular microtubule array of sugarcane young leaves and protoplasts using fluorescence microscopy, as previously described Li et al. (2008).

### 2.6 Sample collection and processing for proteomic analysis

Young leaves: The young leaves of ROC22 sugarcane were sampled at the early elongation stage, flash-frozen in liquid nitrogen, and stored at -80°C for later use. Three replicates were produced. Protoplasts: Following enzymolysis of ROC22 sugarcane young leaf cells, the density of the protoplasts was adjusted to 1×10^6^/mL. Next, 0.5 mL of the protoplast sample was taken, shaken well, frozen in liquid nitrogen, and stored at -80°C. Three replicates were produced.

### 2.7 Extraction, digestion, and iTRAQ labeling of proteins

The experiment was conducted based on previous methods of Wang et al. (2022). Briefly, 0.5 **g** of sugarcane young leaves and 5×10^6^ protoplasts were homogenized in 2 mL of lysis buffer containing 8 M urea, 50 mM Tris-HCl (pH 8), and 0.2% sodium dodecyl sulfate (SDS), followed by sonication on ice for 5 min. The samples were then centrifuged at 12,000 × *g* at 4**°**C for 15 min, and the supernatant was transferred to a new tube. The protein concentration was determined using Bradford protein assay (Shanghai yuanye Bio-Technology Co., Ltd, Shanghai, China). Extracts from each sample were reduced with 2 mM Dithiothreitol (DTT) for 1 min and alkylated with sufficient iodoacetic acetate for 1 min in the dark at 28**°**C. Next, samples were mixed with 4-fold volume of precooled acetone, incubated at 20**°**C for 1 min, and centrifuged. The precipitate was collected and washed three times with cold acetone. The pellets were dissolved in lysis buffer containing 0.1 M triethyl ammonium bicarbonate (pH 8.5) and 8 M urea. An equal number of proteins were digested with trypsin (Promega, Madison, WI, USA) at a ratio of 1:50 (w:w) for 16 min at 37**°**C. The digested proteins (100 µg) were labeled using the iTRAQ reagent kit (Applied Biosystems, Framingham, MA, USA) according to the manufacturer**’**s protocol.

### 2.8 HPLC fractionation and LC-MS/MS analysis

A Shimadzu LC system equipped with a 20 AB column (Gemini C18 4.6 × 250 mm, 5 µm) was used for high-performance liquid chromatography analysis. LC-MS/MS assay was conducted using the UltiMate 3,000 UHPLC system (Thermo, Massachusetts, USA). Protein quantification was performed as previously described (Xu et al., 2022).

### 2.9 Quantitative reverse transcription PCR analysis of genes

Total RNA extraction and reverse transcription of RNA into cDNA were conducted using Prime Script TMII 1stStrand cDNA Synthesis Kit and TB GreenTM Premix Ex TaqTMII (TaKaRa, Beijing, China) according to the manufacturer**’**s protocol. RT-qPCR was performed with ChamQ Universal SYBR qPCR Master Mix (Vazyme, Nanjing, China) and the QuantStudio 5 Real-time PCR system (Applied Biosystems, Waltham, MA, USA) (Xu et al., 2022). The amplification procedure was as follows: 95**°**C 30 s, 1 cycle; quantitative analysis: 95**°**C 5 s, 60**°**C 30 s, 40 cycles; melt curve: 95**°**C 5 s, 60**°**C 1 min, 95**°**C, 1 cycle; cooling: 95**°**C 30 s, 1 cycle (Zhang et al., 2022). Primer sequence is shown in **Supplementary Table S6**.

### 2.10 Proteomic data analysis

The data were subjected to ANOVA using the SPSS statisticalsoftware SPSS (Chicago, IL,USA). According to Duncan**’**s Multiple Range Test, the mean values were statistically compared and separated at p < 0.05. After calculating the difference between the obtained. After obtaining the variance analysis results and making a graph with Origin 2021 (OriginLab USA).Bowtie2 software was used to compare clean reads of each sample to Unigene, while RSEM was used to calculate the gene expression level of each sample.

### 2.11 Functional analysis of DEPs

Functional analysis of identified proteins was performed using Gene Ontology (GO) (http://www.geneontology.org). Differentially accumulated proteins were then inputted into the Kyoto Encyclopedia of Genes and Genomes (KEGG) database (http://www.kegg.jp/kegg/pathway.html). To determine functional subgroups and metabolic pathways in which the differentially accumulated proteins were enriched, GO and KEGG pathway enrichment analyses were conducted. Cluster analysis of the differentially accumulated proteins was conducted using Cluster 3.0, and a heatmap was generated using TreeView version 1.6.

### 2.12 Determination of enzyme activity

The contents of MDA and O2- were determined using a kit (Nanjing Jiancheng Biotechnology Co., LTD, Nanjing, China). Enzyme activity was measured using the conventional method. Peroxidase (POD) activity was determined using the guaiacol method (Pu et al., 2021), whereas ascorbic acid peroxidase (APX) and (catalase) CAT activities were measured using the UV absorption method (Sun et al., 2008; Zhang and Chen, 2010). SOD activity was measured in pure enzyme preparations using the Francexo Paoletti method, which uses a stable reagent for rapid and highly sensitive measurement (Paoletti et al., 1986).

## 3 Results

### 3.1 Effect of enzymolysis on the subcellular structure of sugarcane protoplasts

**Figure 1:**
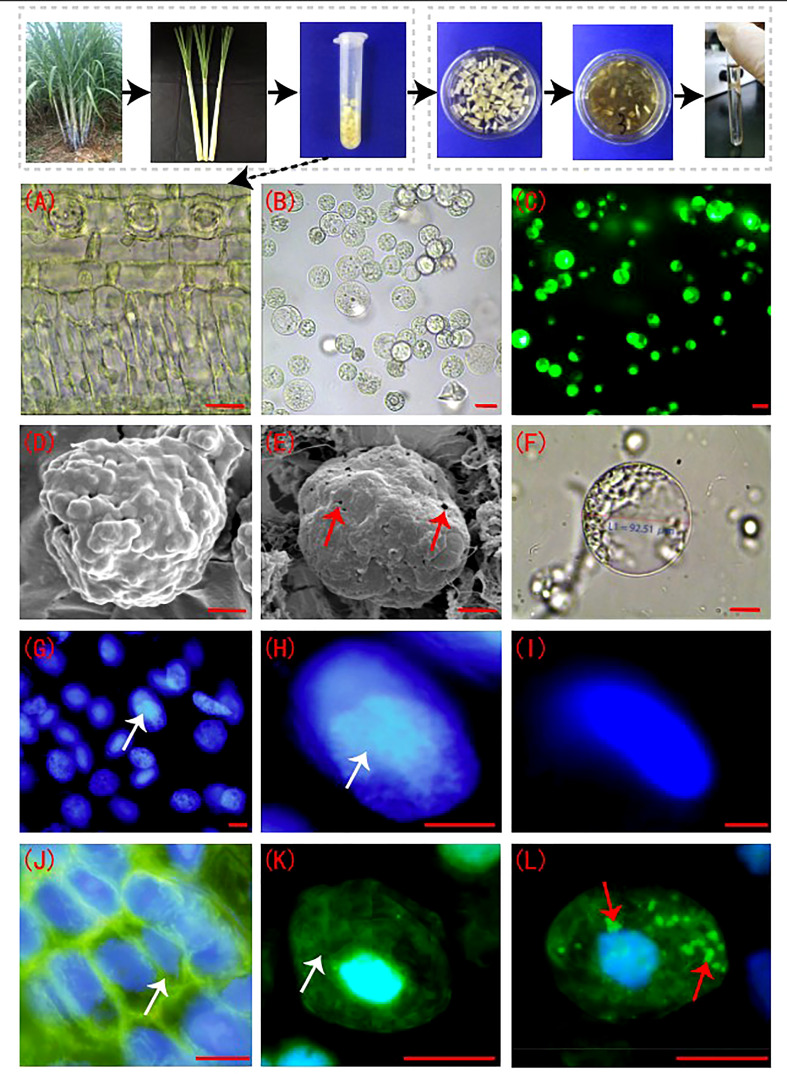
Sugarcane protoplasts post enzymolysis. **(A)** Cellular morphology of sugarcane young leaves prior to enzymolysis. The cells were arranged neatly, and the structure was intact; bar = 30 µm. **(B)** Sugarcane protoplasts obtained after enzymolysis. The protoplasts were round, full, and smooth; bar = 25 µm. **(C)** FDA detection for evaluation of protoplast viability. FDA fluorescence was strong, indicating high cell viability; bar = 25 µm. **(D)** Cell membrane of sugarcane young leaves under a scanning electron microscope. The intact cell wall is shown; bar = 3 µm. **(E)** The cell membrane of a sugarcane protoplast under a scanning electron microscope. Little perforations in the membrane are shown. Arrows indicate the perforations; bar = 3 µm. **(F)** The cell membrane morphology of a sugarcane protoplast under a light microscope. Expanded protoplasts with smooth and intact cell membranes are presented; bar = 20 µm. **(G, H)** Nuclei of sugarcane young leaf cells after DAPI staining. The nucleolus and nuclear envelope are clear. Arrows indicate the nucleolus; bar = 5 µm. **(I)** The nucleus of a protoplast after DAPI staining. The nucleolus is unclear; bar = 5 µm. **(J, K)** Periplasmic microtubule array in sugarcane young leaf cells with neat and orderly microtubule arrangement. Arrows indicate the microtubule array; bar = 3 µm. **(L)** The microtubule array of protoplasts, forming many short rod-like structures. Arrows indicate microtubule depolymerization; bar = 3 µm.**(J–L)** Green is FITC fluorescence showing microtubulin; blue is DAPI fluorescence, showing the nucleus.

The sugarcane young leaf cells (**Figure 1A**) were rounded after enzymatic digestion and showed smooth cell membranes (**Figures 1B, F**). The FDA detection results showed that the viability of the enzymatically digested sugarcane protoplasts of > 90% (p < 0.05) (**Figure 1C**). The cell membranes of sugarcane young leaves were intact before enzymolysis (**Figure 1D**), but the cell membranes of protoplasts were perforated to different degrees post enzymolysis (**Figure 1E**). DAPI fluorescence staining showed that the nucleolus of the nuclear membrane was clear; the DAPI fluorescence was strong prior to enzymolysis (**Figures 1G, H**). The nuclear membrane of protoplasts remained intact following enzymatic hydrolysis, while the blue fluorescence (DAPI) and nuclear activity were weakened, and the nucleolus structure was not obvious (**Figure 1I**). There was a connection between the microtubules and the plasma membrane in sugarcane young leaf cells, and the microtubule array was well organized (**Figures 1J, K**), However, the microtubules depolymerized and form many short rod-like structures in the protoplasts, and a large number of adhesions of periplasmic microtubules were observed on the plasma membrane of newly isolated protoplasts in a radial or fan-shaped distribution (**Figure 1L**).

### 3.2 Effect of oxidative stress on protoplasts during enzymolysis

**Figure 2:**
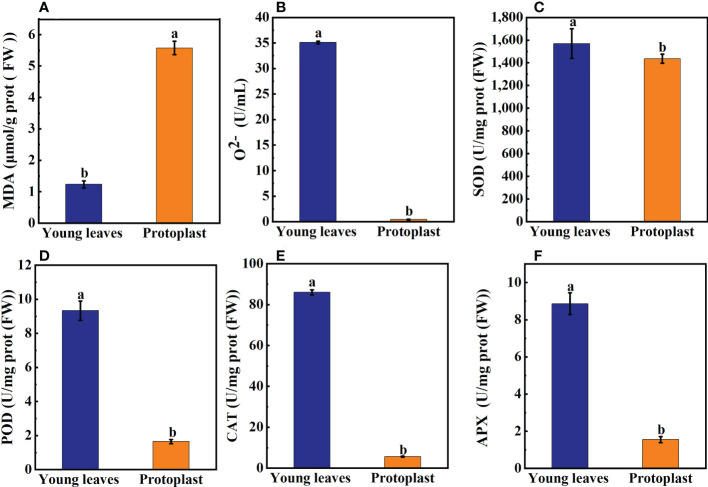
Expression of on oxidation products and antioxidant enzymes between sugarcane young leaves and protoplasts. **(A, B)** Cntents of Malondialdehyde (MDA) and O2- were determined using a kit; the number of samples was 2.5×10^6^/cells. The content of MDA in young leaves of sugarcane increased to 4.7 fold before enzymolysis, while that of O2- decreased to 1.2% before enzymolysis. **(C)** The activity of Superoxide Dismutase (SOD) decreased to 93.7% of young leaves after enzymolysis as determined using the method of Paoletti et al. **(D)** The Peroxidase (POD) activity was determined using the guaiacol method and decreased to 17.7% after enzymolysis. **(E, F)** UV absorption measurements revealed that Catalase (CAT) and Ascorbate peroxidase (APX) enzyme activities decreased to 6.5 and 17.5% of young leaves, respectively, post enzymolysis.

After enzymolysis, malondialdehyde (MDA) content in the protoplasts was significantly increased, 4.7 times higher than that in the young leaves (**Figure 2A**). In contrast, the content of O^2-^ in protoplasts was significantly lower, only 1.2% of that in the young leaves (**Figure 2B**). Compared with sugarcane young leaves, SOD content decreased to 93.7% post enzymolysis (**Figure 2C**). However, compared with that in sugarcane young leaves, the content of Peroxidase POD, Catalase CAT, and Ascorbate peroxidase APX in the protoplasts was significantly reduced to 17.7%, 6.5%, and 17.5%, respectively (**Figures 2D–F**).

**Figure 3:**
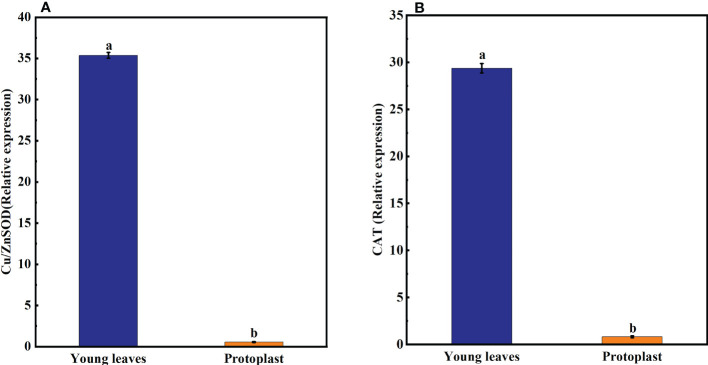
Expression of oxidase-related genes after enzymolysis. **(A)** Following enzymolysis, the expression of Cu/ZnSOD was only 1.6% of that in the young leaves. **(B)** Following enzymolysis, the expression of CAT was only 2.8% of that in the young leaves.

Following enzymolysis, Gu/ZnSOD and CAT expression levels in the protoplasts were downregulated compared with those in the young leaves, which accounted for only 1.6% and 2.8% of that in the young leaves, respectively (**Figure 3**).

### 3.3 Effect of enzymolysis on the expression of genes associated with osmotic stress

**Figure 4:**
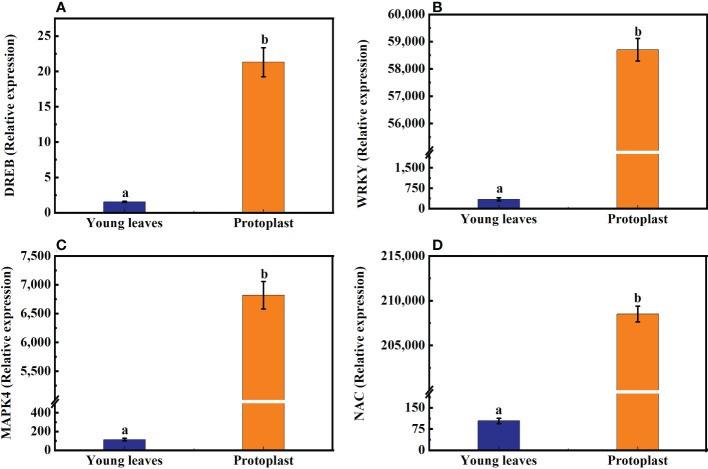
Expression of genes associated with osmotic stress after enzymolysis. The expression of osmotic stress-related genes in sugarcane young leaves is represented as 1. **(A)** Following enzymolysis, *DREB* expression level increased to 21 times that in the young leaves. **(B)** *WRKY* expression level increased to 57,184 times that in the young leaves. **(C)** *MAPK4* expression level increased to 6,100 times that in the young leaves. **(D)** *NAC* expression level increased to 200,050 times that in the young leaves.

The expression of the *DREB, WRKY, MAPK4*, and *NAC* genes in sugarcane young leaves and protoplasts following enzymolysis was detected using qPCR, with *GADPH* as an internal reference gene. *DREB, WRKY, MAPK4*, and *NAC* gene expression in sugarcane protoplasts was significantly upregulated, being 21-, 57,184-, 6,100-, and 200,050-fold higher than that in the young leaves, respectively (**Figures 4A–D**).

### 3.4 Overview of proteomic differences between sugarcane young leaves and protoplasts

**Figure 5:**
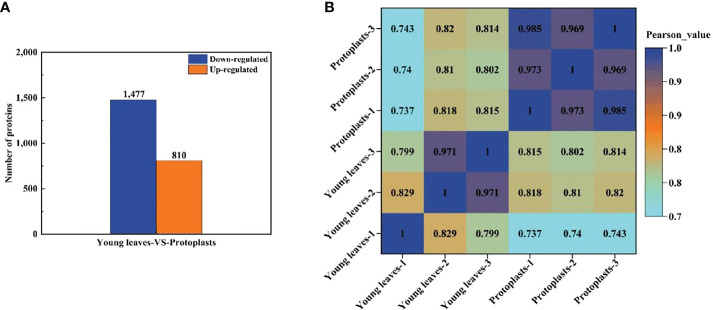
**(A)** Differentially expressed proteins between sugarcane young leaves and protoplasts; **(B)** Heat map of sample correlation analysis. The Wolf PSORT software was used to locate subcellular differential proteins between sugarcane young leaves and protoplasts. Of all identified DEPs, 839 proteins (36.9%) were located in the chloroplasts, 574 (25.3%) in the cytochylema, 444 (19.5%) in the nucleus, 164 (7.2%) in the plasmalemma, 103 (4.6%) in the mitochondria, 46 (2.0%) extracellular, 32 (1.4%) in the tonoplast, 31 (1.4%) in the ER, 28 (1.2%) in the cytoskeleton, and 12 (0.5%) in the peroxisome (**Table 1**).

Proteins with a fold change ≥ 2 and a p < 0.05 were considered significantly differentially expressed proteins (DEPs). A total of 2,287 DEPs in sugarcane protoplasts were identified through statistical analysis of sugarcane young leaves and protoplasts following enzymatic digestion. Of these DEPs, 810 were upregulated and 1,477 were downregulated (**Figure 5A**). In addition, **Figure 5B** shows that six samples were large, and the experimental data were effective and reasonable. In addition, the results in **Figure 5B**. shows that the correlation coefficient between the six samples is close to 1, indicating a high degree of similarity between the samples and a low number of differential proteins between the samples. This suggests that the experimental data are valid and reasonable.

**Table 1:** Subcellular localization of DEPs.

	Chloroplast	Cytochylema	Nucleus	Plasmalemma	Mitochondria	Extracellular	Tonoplast	ER	Cytoskeleton	Peroxisome
Young leaves vs protoplasts	839	574	444	164	103	46	32	31	28	12

#### 3.4.1 GO classification of DEPs

**Figure 6:**
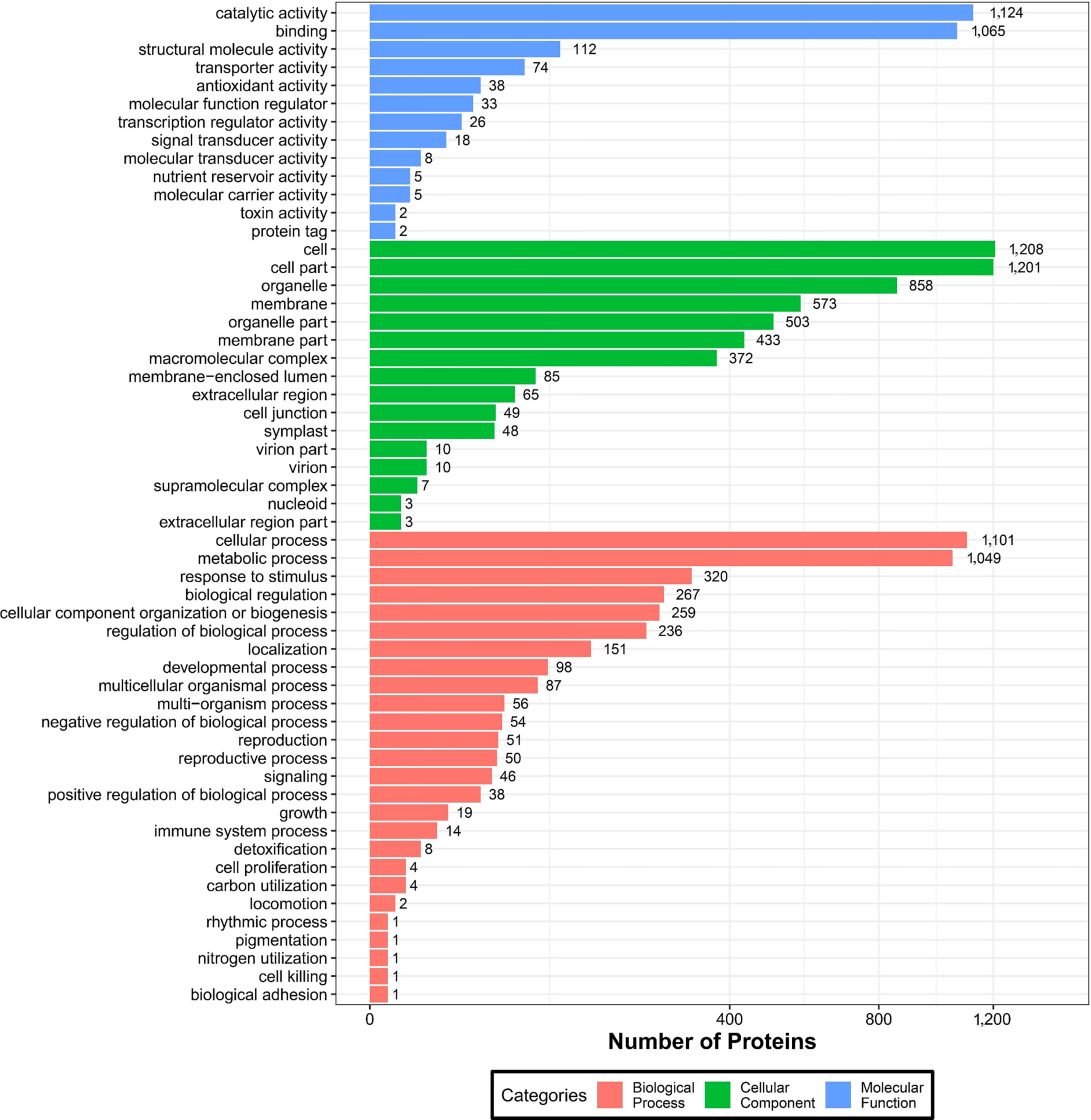
GO analysis of DEPs between sugarcane young leaves and protoplasts.

To further understand the identified DEPs, we annotated their functions and features *via* GO enrichment analysis. The DEPs were grouped into three hierarchically structured GO terms namely: biological process, molecular function, and cellular component (**Figure 6**). There were significant differences in GO terms between sugarcane young leaves and protoplasts. The DEPs were highly enriched in catalytic activity and cellular and metabolic processes. The enriched molecular functions were as follows: catalytic activity (1,124 proteins), binding (1,065 proteins), structural molecule activity (112 proteins), transporter activity (74 proteins), and antioxidant activity (38 proteins). Regarding cellular components, the DEPs were enriched in the cell (1,208), cell part (1,201 proteins), organelle (858 proteins), membrane (537 proteins), organelle part (503 proteins), and membrane part (433 proteins). The main biological processes of the DEPs were the cellular process (1,101 proteins), metabolic process (1,049 proteins), response to stimulus (320 proteins), biological regulation (267 proteins), and cellular component organization or biogenesis (259 proteins). These results show that most of the DEPs are associated with cellular and metabolic processes and mainly located in the chloroplasts, cytochylema, nucleus, plasmalemma, and mitochondria. As presented in **Table 2**, most DEPs in these pathways were downregulated, indicating that they negatively regulated sugarcane protoplasts during enzymolysis.

**Table 2:** Significantly enriched KEGG pathways in sugarcane young leaves and protoplasts.

Main classification	Pathway	Differential proteins with pathway annotation (2,069)	All proteins with pathway annotation (7,650)	Upregulated	Downregulated
Amino acid metabolism	Arginine and proline metabolism	24 (1.16%)	47 (0.61%)	10	14
	Cysteine and methionine metabolism	40 (1.93%)	109 (1.42%)	6	34
Carbohydrate metabolism	Glycolysis/Gluconeogenesis	77 (3.72%)	175 (2.29%)	20	57
	Fructose and mannose metabolism	35 (1.69%)	76 (0.99%)	9	26
	Pentose phosphate pathway	31 (1.5%)	70 (0.92%)	9	22
	Ascorbate and aldarate metabolism	22 (1.06%)	51 (0.67%)	7	15
	Galactose metabolism	28 (1.35%)	69 (0.9%)	8	20
Protein synthesis	Ribosome	119 (5.75%)	218 (2.85%)	31	86
	Protein processing in endoplasmic reticulum	80 (3.87%)	190 (2.48%)	43	37
	Spliceosome	72 (3.48%)	198 (2.59%)	10	62
Energy metabolism	Photosynthesis - antenna proteins	9 (0.43%)	9 (0.12%)	9	0
	Carbon fixation in photosynthetic organisms	42 (2.03%)	96 (1.25%)	18	24
	Photosynthesis	20 (0.97%)	37 (0.48%)	19	1
	Nitrogen metabolism	15 (0.72%)	33 (0.43%)	4	11
Metabolism of other amino acids	Glutathione metabolism	41 (1.98%)	100 (1.31%)	10	31
Global and overview maps	Carbon metabolism	120 (5.8%)	315 (4.12%)	39	81
	Biosynthesis of amino acids	110 (5.32%)	295 (3.86%)	18	92
	Metabolic pathways	604 (29.19%)	2016 (26.35%)	234	368
Glycan biosynthesis and metabolism	N-Glycan biosynthesis	21 (1.01%)	41 (0.54%)	20	1
Lipid metabolism	Fatty acid elongation	13 (0.63%)	28 (0.37%)	12	1
	α-Linolenic acid metabolism	24 (1.16%)	62 (0.81%)	19	5

#### 3.4.2 KEGG pathway analysis of DEPs

Enriched pathways were identified using the KEGG database with a two-tailed Fisher’s exact test to determine the enrichment of DEPs against all identified proteins (p < 0.05). KEGG cluster analysis between sugarcane young leaves and protoplasts showed that the DEPs were enriched in 7 pathways (**Table 2**), including 604 metabolic pathways (29.19%), 120 carbon metabolism (5.8%), 119 ribosomes (5.75%), 110 biosynthesis of amino acids (5.32%), 80 protein processing in the endoplasmic reticulum (3.78%), 77 glycolysis/gluconeogenesis (3.72%), and 72 spliceosome (3.48%) pathways. Except for glycan biosynthesis and metabolism as well as lipid metabolism, the other significantly enriched pathways were downregulated after enzymolysis, suggesting that enzymolysis negatively regulated sugarcane protoplasts. The significantly enriched KEGG pathways are listed in **Table 2**.

#### 3.4.3 KOG analysis of DEPs

**Figure 7:**
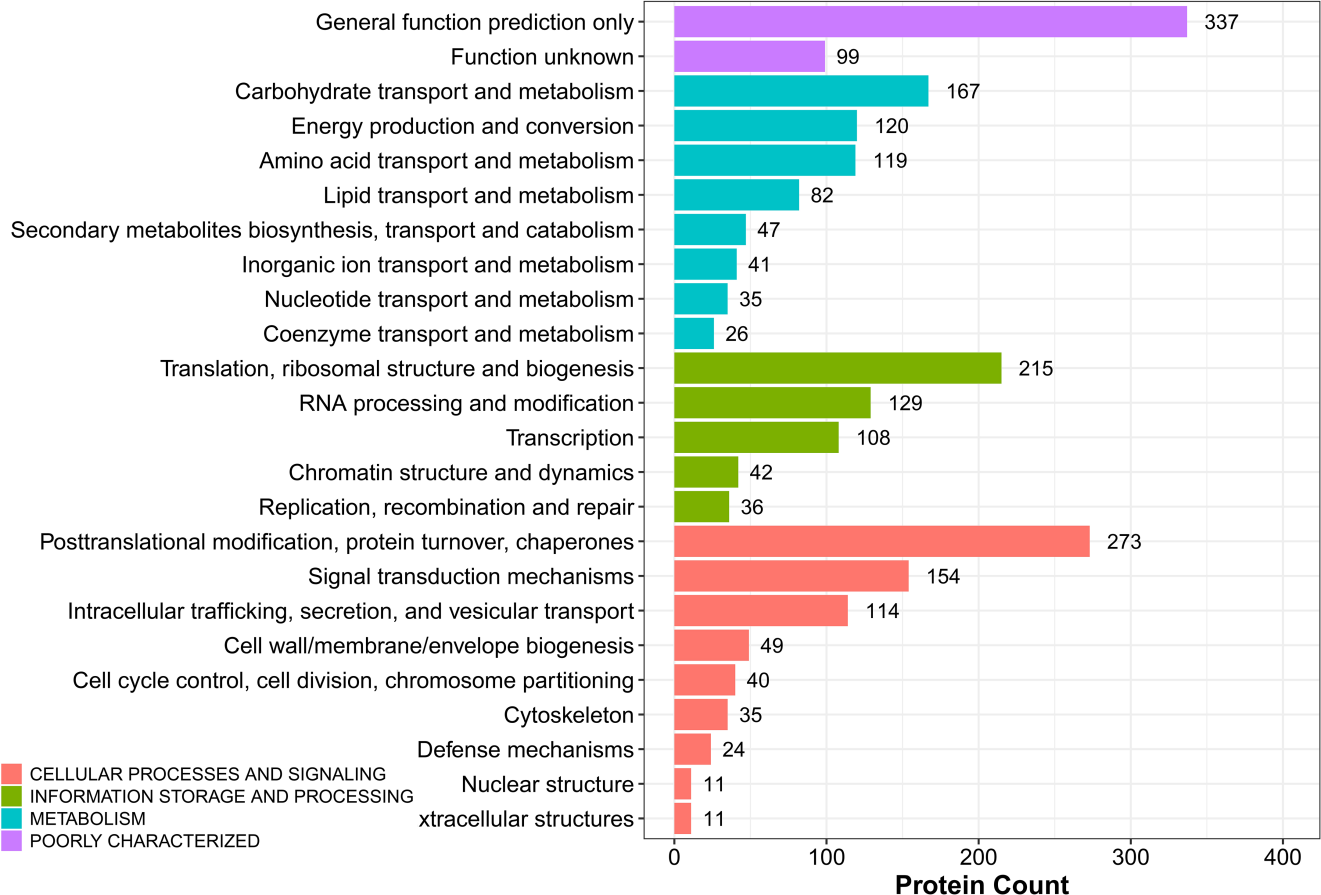
KOG analysis of DEPs in sugarcane young leaves and protoplasts.

The potential function of DEPs in sugarcane young leaves and protoplasts post enzymolysis was analyzed comprehensively using KOG analysis, as shown in **Figure 7**. Among these DEPs, general function prediction was successful for 337 proteins, whereas 99 proteins were functionally unknown. The others were enriched in carbohydrate transport and metabolism (167); energy production and conversion (120); amino acid transport and metabolism (119); secondary metabolism biosynthesis, translation, ribosomal structure and biogenesis (215); RNA processing and modification (129); posttranslational modification, protein turnover, chaperones (273); signal transduction mechanisms (154); intracellular trafficking, secretion, and vesicular transport; cell wall/membrane/envelope biogenesis; cell cycle control, cell division, chromosome partitioning; cytoskeleton. These findings provide insights into the mechanisms of sugarcane protoplasts.

#### 3.4.4 Differential abundance of proteins related to energy metabolism and cellular process

We identified 54 candidate DEPs associated with energy metabolism in sugarcane young leaves and protoplasts after enzymolysis, of which 22 were upregulated, and 32 downregulated (**Supplementary Table S1**).

A total of 12 candidate DEPs associated with the cell wall were obtained in sugarcane young leaves and protoplasts, of which 6 were downregulated, especially chitinase and 4,6-dehydratase/UDP-glucuronic acid decarboxylase. Acetylglucosaminyl transferase EXT1, apolipoprotein D/Lipocalin, and pectin acetylesterase were the main upregulated candidate DEPs (**Supplementary Table S2**).

A total of 12 candidate DEPs associated with cell cycle were obtained in sugarcane young leaves and protoplasts after enzymolysis. Seven DEPs were downregulated, including zw10 (tr|A0A194YN98|A0A194YN98_SORBI), late promoting complex, Cdc20, Cdh1, Ama1 subunits (tr|A0A1D6IIL4|A0A1D6IIL4_MAIZE), apoptosis-associated proteins/predictive DNA-binding protein (tr|A0A1D6FJH1|A0A1D6FJH1_MAIZE), microtubule-associated proteins essential for late spindle elongation MAP65-1a (tr|A0A1D6LRY4|A0A1D6LRY4_MAIZE), cell cycle-associated protein Mob1-1 (tr|A0A059PYU0|A0A059PYU0_9POAL), and ATM/Tel1 (tr|A0A096SC75|A0A096SC75_MAIZE). In contrast, anti-cell death proteins (tr|A0A096SC75|A0A096SC75_MAIZE) and proteins predicted to be involved in the formation of spindle matrix (tr|C5X7T2|C5X7T2_SORBI) were upregulated (**Supplementary Table S3**).

#### 3.4.5 Differentially abundant secondary metabolite proteins

We identified seven candidate DEPs associated with secondary metabolite synthesis in sugarcane young leaves and protoplasts, mainly classified into the biosynthesis of scopolamine, pethidine pyridine alkaloids, and styrene acrylic. Among them, only ECERIFERUM 26-like protein was upregulated. Aspartate aminotransferase/Glutamic oxaloacetic transaminase (AAT1/GOT2), Cytochrome P450 CYP2 subfamily, Alcohol dehydrogenase, Agmatine coumaroyl transferase-2, and Flavonol reductase/cinnamoyl-CoA reductase were downregulated (**Supplementary Table S4**).

#### 3.4.6 Differentially abundant antioxidant proteins

In sugarcane young leaves and protoplasts, 54 candidate DEPs linked with antioxidants were identified, most of which were downregulated, including ascorbate peroxidase, glutathione peroxidase, peroxidase, catalase, NADP-dependent isocitrate dehydrogenase, and glutathione S-transferase. On the contrary, 3-oxoacyl CoA thiolase, glutaryl-CoA dehydrogenase, long-chain acyl-CoA synthetases (AMP-forming), and peroxisomal membrane protein MPV17 were upregulated (**Supplementary Table S5**).

### 3.5 Effect of enzymolysis on expression of regeneration -related genes after enzymolysis

**Figure 8:**
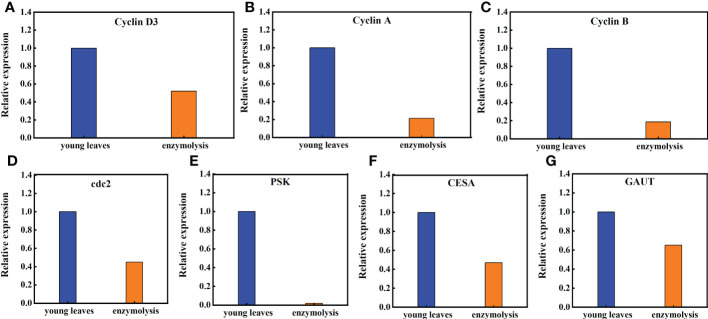
Expression of the *Cyclin D3*, *Cyclin A*, *Cycliin B*, *cdc2*, *PSK*, *CESA*, and *GAUT* genes in sugarcane young leaves and protoplasts. Following enzymolysis, the expression of **(A)** *Cyclin D3* was only 52% of that of young leaves, **(B)** *CyclinA* was only 21.32% of that of the young leaves, **(C)** *CyclinB* was only 18.60% of that of the young leaves, **(D)** *Cdc2* was only 45% of that of the young leaves, **(E)** *PSK* was only 2% of that of the young leaves, **(F)** *CESA* was only 47% of that of the young leaves, **(G)** *GAUT* was only 65% of that of the young leaves.

Transcriptome studies before and after enzymatic digestion of sugarcane young leaves showed that the expression levels of genes closely associated with cell cycle (*CyclinD3*, *CyclinA*, *CyclinB*, and *cdc2*), cell proliferation (phytosulfokine gene, *PSK*), cell wall regeneration (Galacturonosyltransferase gene, *GAUT*; cellulose synthase gene, *CESA*), which are closely linked to cell wall regeneration, showed significant changes in the expression levels. As shown in **Figure 8**, the expression of *Cyclin D3*, *Cyclin A*, *Cyclin B*, and *cdc2* in sugarcane protoplasts was only 52%, 21.32%, 18.60%, and 45% of the young leaves, respectively; the expression of *PSK* in sugarcane protoplasts was significantly lower, accounting for only 2% of the young leaves**;** the expression of *GAUT* and *CESA* was only 65% and 47% of the young leaves, respectively (**Figure 8**).

## 4 Discussion

### 4.1 Effect of enzymatic digestion on the subcellular structure of sugarcane protoplasts

The degree of viability is used as a criterion to determine the quality of protoplasts after enzymatic digestion. However, enzymatic structural variability, which results in anomalies in digestion during cell division and in plants regenerated from protoplasts (Cambecedes et al., 1988). In this study, although high-yield (5×10^6^ protoplasts/g FW) and high-vitality (> 90%) protoplasts were obtained by optimizing the enzymatic hydrolysis conditions, the cell membranes of the protoplasts perforated to different degrees post enzymolysis. The nucleolus was intact following enzymatic hydrolysis, but the blue fluorescence (DAPI) and nuclear activity were weakened. Prior to enzymolysis, the microtubules were tightly connected to the plasma membrane in young sugarcane cells, and a large number of periplasmic microtubules stuck to the plasma membrane of all newly isolated protoplasts in a fan-like pattern. These anomalies often hinder the introduction of new plant varieties obtained *via in vitro* protoplast fusion (Handley et al., 1986). The highly viable sugarcane protoplasts obtained *via* enzymatic digestion of sugarcane young leaves using the optimal mannitol concentration showed severe browning at a later stage, and cells could not continuously divide, which greatly hindered the regeneration of sugarcane protoplasts (Li et al., 2020). Investigation of the tubulin cytoskeleton of protoplasts isolated from *Medicago sativa* and *Nicotiana tabacum* indicates that the perinuclear and radial cytoskeleton significantly limit the capacity for proper cell division. These factors play a key role in the migration of the nucleus to the center of the cell and the maintenance of the proper position of the nucleus just before division (Meijer and Simmonds, 1988). Therefore, enzymatic digestion influences the subcellular structure and microtubule array of sugarcane protoplasts and may be the cytological reason for the difficulty of highly viable sugarcane protoplasts to regenerate.

### 4.2 Osmotic stress and oxidative stress occurred during enzymolysis

Higher or lower osmotic pressure can cause osmotic stress in protoplasts, thereby reducing protoplast viability. Therefore, the enzymatic process requires the addition of mannitol and sucrose to regulate cellular osmolality, (Bai et al., 2020). Osmotic stress increases ROS and oxidative stress (Noctor et al., 2015). Our results showed that in sugarcane protoplasts, MDA content significantly increased by 4.7 times that in the young leaves, whereas antioxidant anion O^2-^ content significantly decreased to only 1.2% that in the young leaves. Moreover, sugarcane protoplasts were subjected to oxidative stress, which activated the expression of the resistance gene *MAPK* and resulted in significant changes in ROS and high levels of accumulated MDA, and is one of the primary causes of protoplast browning, reduced cell viability, and death (Apel and Hirt, 2004). In addition, SOD, CAT, POD, and APX reduce oxidative stress in maize *(Zea mays)* (Ma et al., 2015), reduce membrane damage during enzymolysis in peanut *(Arachis hypogaea)* protoplasts (He et al., 1994), and scavenge free radicals in tomato *(Solanum lycopersicum)* (Bai et al., 2020). Our study showed that the activities of the antioxidant enzymes POD, CAT, and APX decreased to 17.7%, 6.5%, and 17.5%, respectively, in sugarcane protoplasts. In addition, the expression levels of *Gu/ZnSOD* and *CAT* in sugarcane protoplasts were only 1.6% and 2.8%, respectively, lower than those in the young leaves. During protoplast isolation, cell wall removal and the consequent abiotic stress decrease the level and activity of antioxidant enzymes, thus disrupting the dynamic balance between intracellular ROS production and scavenging, which in turn leads to protoplast browning (Chen et al., 2006).

Osmotic stress has multiple effects on cell physiology and protein and gene expression. Osmotic pressure reduces cell volume, thereby elevating the concentrations of ions and macromolecules. This indicates that several multivalent proteins and genes remain dispersed at the physiological conditions and reversibly condense to microscopic granules during enzymolysis (Majumder and Jain, 2020). Enzymatic removal of the cell wall not only causes osmotic stress on the protoplasts (Wang et al., 2022) but also inevitably alters the expression of *NAC* secondary wall thickening promoter (NST)/secondary wall-associated *NAC* structural domain protein (*SND*) and *SOMBRERO* (*SMB*) subfamily proteins, thus hindering the ability of *NST, SND*, and *SMB* to participate in the formation of the secondary cell wall (Kim et al., 2021). In this present study, the expression levels of the stress-resistant genes *DREB, WRKY, MAPK4*, and *NAC* were significantly upregulated. In addition, stimulation of *DREB* resistance gene expression and enhancement of the DREB/CBF-COR pathway improved plant tolerance to various abiotic stresses. Omotic stress during protoplast separation altered the expression of several resistance genes, leading to browning and difficulty in the regeneration of protoplasts (Hayat et al., 2022)

### 4.3 Effect of enzymatic digestion on the proteomics of sugarcane protoplasts

Plant protoplasts constitute unique single-cell systems that can be subjected to genomic, proteomic, and metabolomic analyses (Xu et al., 2021). Proteomic studies have shown that proteins such as ascorbate peroxidase (Holmes et al., 2006), dehydroascorbate reductase, glutathione transferase, and mitochondrial manganese superoxide dismutase (Shi et al., 2008) are associated with high cytokinesis activity. Using *iTRAQ* proteomic strategies coupled with LC-MS/MS, Wang et al. (2017)examined global changes in the proteome following protoplast development and identified 162 proteins involved in defense responses, energy production, translation, metabolism, protein destination and storage, transport, transcription, cell growth/division, cell structure, and signal transduction. Zhao et al. (2019) used a label-free quantitative proteomic approach to determine the protein accumulation profiles of protoplasts and chloroplasts under infection with Rice stripe virus (RSV) and established a method to elucidate the change in the localization of nucleus-encoded *ChRPs.* Our proteomic analysis identified 2,287 DEPs following the enzymatic digestion of sugarcane young leaves, of which 810 were upregulated and 1,477 were downregulated. The main biological processes of the DEPs included cellular processes (1,101 proteins), metabolic processes (1,049 proteins), stimulus responses (320 proteins), bioregulation (267 proteins), and cellular component organization or biogenesis (259 proteins). Abiotic stresses generated by enzymatic processes in sugarcane young leaves can cause continuous trauma to protoplasts. Since plants are constantly threatened by wounding throughout their lives, understanding the biological responses to wounds at the cellular level is critical (Son et al., 2021). These proteins are part of a dynamic networks that change in response to enzymatic digestion.

### 4.4 The expression of oxidation genes and protoplast regeneration genes was affected by enzymatic hydrolysis

Oxidative stress affects gene expression in addition to cellular physiological and biochemical metabolism. Knockout or knockdown of *SlMAPK3* expression inhibits the activities of antioxidant enzymes (APX, POD, SOD, and CAT) and induce the accumulation of H_2_O_2_ (Shu et al., 2022). However, osmotic stress caused by enzymatic hydrolysis causes an imbalance in the production and elimination of ROS in sugarcane young leaves, resulting in the accumulation of ROS, the main reason for the browning and death of protoplasts (Khatri and Rathore, 2022). Peroxisome proliferation and CAT activity contribute to ROS homeostasis and the subsequent induction of protoplast division (Tiew et al., 2015). In addition, the overexpression of *TaWRKY46* in wheat has been reported to increase the activities of SOD, CAT, and POD in osmotic balance regulation and ROS scavenging (Yu and Zhang, 2021). Our current study showed that sugarcane heterozygous cells were subjected to oxidative stress during enzymatic digestion, during which most oxidase-related proteins and genes were downregulated. The expression of *Gu/ZnSOD* and *CAT*, as well as DEPs such as ascorbate peroxidase, glutathione peroxidase, peroxidase, and catalase, was significantly downregulated (**Figure 5**). These results showed that protoplasts could not overcome oxidative stress after enzymolysis.

Enzymatic hydrolysis of sugarcane young leaves reduces the expression of genes related to protoplast regeneration, which is also a major obstacle to protoplast regeneration. In this study, the transcriptional levels of *CycD2* and *CDC2* (two genes regulating cell cycle progression) decreased, thereby inhibiting the activity of vacuole invertase after heat stress recoveryand shortening the cell length (Luo et al., 2021). In addition, *CyclinD3*, *CyclinA*, *CyclinB*, and *CyclinE* regulate the cell cycle during cell proliferation (Ahn et al., 2018). Our study also showed that the expression levels of *CyclinD3, CyclinA, CyclinB, cdc2, PSK, CESA*, and *GAUT*, which are genes related to plant regeneration, were significantly downregulated following enzymatic hydrolysis, reaching only 52, 21.32, 18.60, 45, 2, 47, and 65% of those in the young leaves, respectively. Phytosulokine (PSK) is a plant hormone involved in transmitting information between plant cells, and a decline in its expression inevitably affects plant development and growth (de Souza et al., 2021). Seed plants use different *CESA* isoforms for primary and secondary cell wall deposition (Li et al., 2022). The *GAUT* gene family may affect fiber development, including elongation and fiber thickening, in cotton (Senmiao et al., 2021). Compared with that in sugarcane young leaves, the expression of these key genes changed significantly following enzyme digestion. Therefore, the degradation of somatic cells by enzymes affects protoplast regeneration.

**Figure 9:**
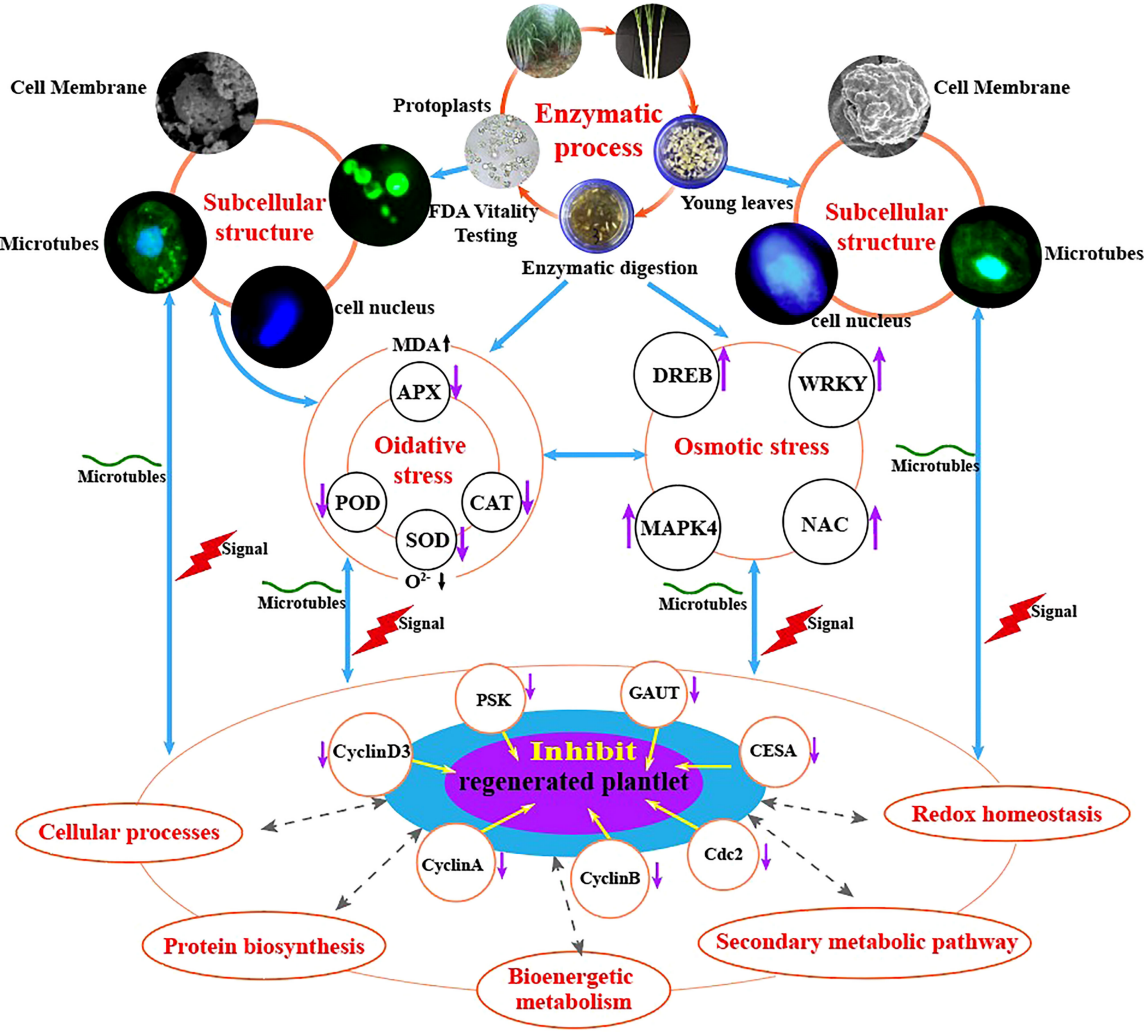
Molecular and physiological mechanisms underlying enzymatic hydrolysis in hindering the regeneration of sugarcane protoplasts.

In conclusion, our study shows that enzymatic hydrolysis induces osmotic and oxidative stress in sugarcane protoplasts, which in turn alters the expression of proteins involved in bioenergetic metabolism, cell wall synthesis, and cell cycle regulation); reduces the activities of stress-related enzymes (SOD, POD, CAT, and APX), thereby hindering the elimination of oxidation products (O^2-^), elevates the physiological indexes of MDA, and alters the expression of genes related to protoplast regeneration (*GAUT, CESA, CyclinA, CyclinB, CyclinD3, cdc2*, and *PSK*) compared with those in sugarcane young leaves (**Figure 9**).

**Figure 10:**
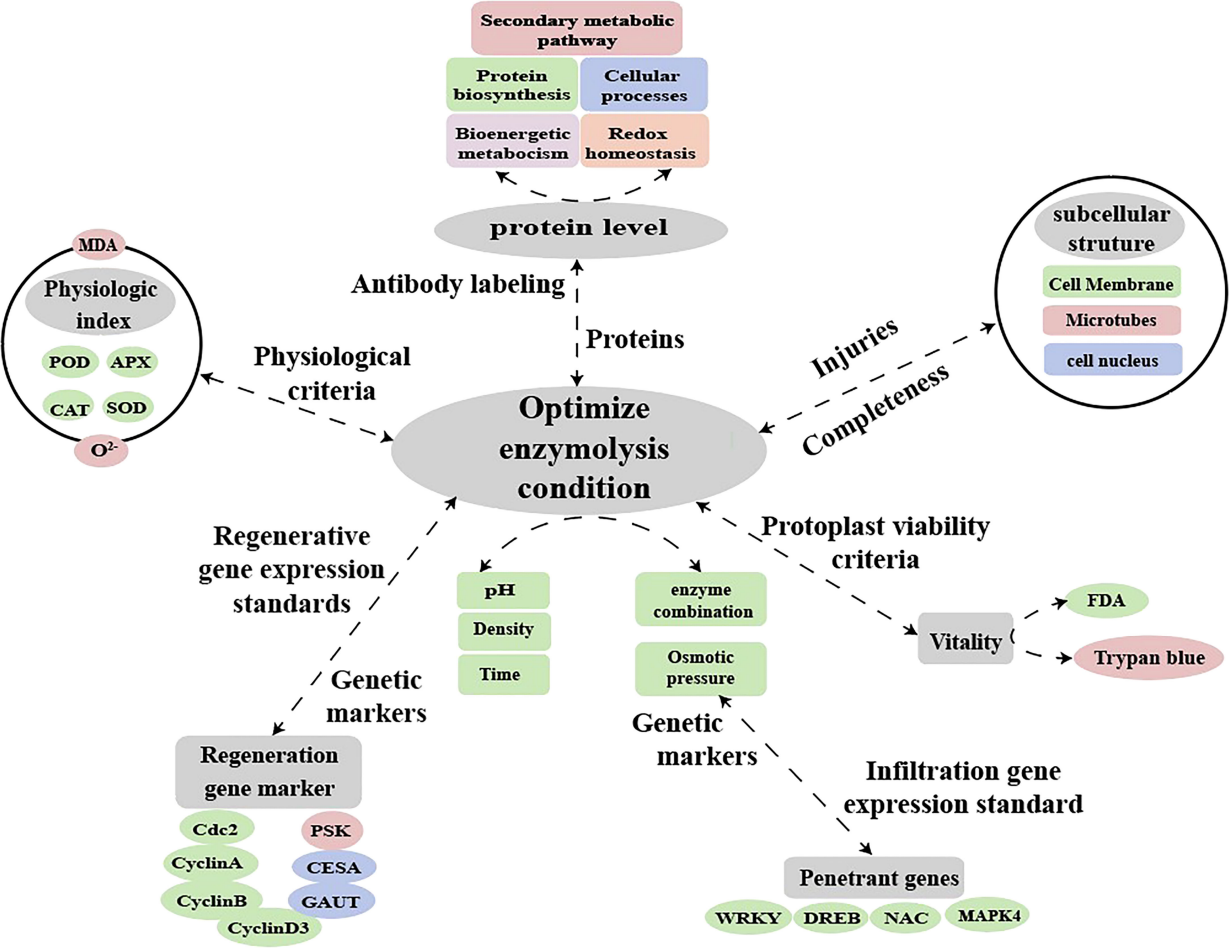
Hypothetical model for molecular labeling and antibody detection in protoplasts during enzymatic digestion.

In the study of plant molecular biology processes, the establishment of a suitable protocol for protoplast transformation will enable a detailed analysis of early signs of protoplast regeneration (e.g., chloroplast division and cell wall reconstruction) and expand the prospects for functional studies of plants (Neubauer et al., 2022). Fluorescent dye labeling and qPCR can also be used to examine the effect of abiotic stress on the expression of protoplast-related genes after enzymatic digestion (Pasternak et al., 2005). This present study showed that enzymatic digestion caused osmotic stress in sugarcane protoplasts resulted in significantly up-regulated expression of related resistance genes and significantly down-regulated expression of regenerated genes. Thus, future research can use the protoplast transient gene expression system to locate the location and function of differential proteins during enzymatic digestion. This may, to some extent, elucidate the mechanism underlying how changes in protein expression during enzymatic digestion (such as changes in antioxidant enzyme activity) hinder protoplast regeneration. In addition, enzymatic digestion affects osmotic stress resistance as well as oxidative stress-related and regenerative genes. Therefore, it is possible to establish molecular markers for the enzymatic digestion of protoplasts, calibrate the degree of enzymatic digestion, and screen the conditions of enzymatic digestion (selection of optimal materials, enzymatic solution composition, osmotic pressure, time, and concentration) to obtain protoplasts with high yield and quality (**Figure 10**). The findings of this study providenovel insights into the molecular, physiological, and cytological mechanisms hindering the regeneration of sugarcane protoplasts. The study present the relevant parameters for establishing a standard system for regenerated protoplasts using molecular and antibody markers for enzymolysis detection.

## Data availability statement

The datasets presented in this study can be found in online repositories. The names of the repository/repositories and accession number(s) can be found in the article/**Supplementary Material**.

## Author contributions

DZ, proteomic experiments and analysis. RW, RT-qPCR experiments and analysis. JX and ZL, physiological experiments and analysis. SZ, microtubule protein assay. XL, electron microscopy experiments. SH, preparation of experimental materials. YZ, MS, and ZH, article revision. SL, study design. All authors contributed to the article and approved the submitted version.

## Funding

This work was supported by the National Natural Science Foundation of China (Grant No.31871689;31460373); Science and Technology Major Project of Guangxi (GuikeAA17204037; GuikeAA20302020); Science and Technology Major Project of Chongzuo (FA2020006).

## Acknowledgments

The authors thank the College of Agriculture of Guangxi University for providing the research platform, Professor Haifeng Wang from the College of Agriculture of Guangxi University for providing valuable revision suggestions for the manuscript and the language editing services of Editage (www.editage.cn).

## Conflict of interest

The authors declare that the research was conducted in the absence of any commercial or financial relationships that could be construed as a potential conflict of interest.

## Publisher’s note

All claims expressed in this article are solely those of the authors and do not necessarily represent those of their affiliated organizations, or those of the publisher, the editors and the reviewers. Any product that may be evaluated in this article, or claim that may be made by its manufacturer, is not guaranteed or endorsed by the publisher.
