# Supplementary material for: An integrated physiology, cytology, and proteomics analysis reveals a network of sugarcane protoplast responses to enzymolysis
Source: Front Plant Sci. 2022 Nov 28;13:1066073. doi: 10.3389/fpls.2022.1066073 (PMC9744229; doi:10.3389/fpls.2022.1066073)
Supplement: Supplementary file 1 [file DataSheet_1.docx]

Supplementary Table S1. Candidate DEPs associated with energy metabolism

| **Protein ID** | **Protein description** | **Difference multiple** |
| --- | --- | --- |
|  |  | **Treated1/ control** |
|  |  | **(Up/down 22/32)** |
| sp\|A1E9S1\|ATPA_SORBI | F0F1-type ATP synthase, alpha subunit | 3.30 |
| tr\|A0A1B6PB20\|A0A1B6PB20_SORBI | 5'-AMP-activated protein kinase, gamma subunit | -2.56 |
| tr\|A0A1B6PK61\|A0A1B6PK61_SORBI | 5'-AMP-activated protein kinase, gamma subunit | -1.92 |
| tr\|A0A3L6E0E5\|A0A3L6E0E5_MAIZE | Acyl carrier protein/NADH-ubiquinone oxidoreductase, NDUFAB1/SDAP subunit | -2.25 |
| tr\|C5Z842\|C5Z842_SORBI | Acylphosphatase | -2.28 |
| tr\|A0A194YJY8\|A0A194YJY8_SORBI | Aldehyde dehydrogenase | 2.41 |
| tr\|A0A1D6ER25\|A0A1D6ER25_MAIZE | Aldehyde dehydrogenase | -3.07 |
| tr\|A0A1Z5REV8\|A0A1Z5REV8_SORBI | Aldehyde dehydrogenase | 2.42 |
| tr\|A0A3L6FWF8\|A0A3L6FWF8_MAIZE | Aldehyde dehydrogenase | 2.52 |
| tr\|A0A1D6NYE7\|A0A1D6NYE7_MAIZE | Aldehyde dehydrogenase | -1.23 |
| tr\|A0A1D6Q1L8\|A0A1D6Q1L8_MAIZE | Aldehyde dehydrogenase | -1.94 |
| tr\|A0A1D6GMM7\|A0A1D6GMM7_MAIZE | Cytochrome b5 | -2.71 |
| tr\|A0A3L6G5V1\|A0A3L6G5V1_MAIZE | Electron transfer flavoprotein, beta subunit | -3.31 |
| sp\|A1E9S1\|ATPA_SORBI | F0F1-type ATP synthase, alpha subunit | 3.30 |
| sp\|A1E9T1\|ATPB_SORBI | F0F1-type ATP synthase, beta subunit | 3.15 |
| tr\|A0A1B6QEH8\|A0A1B6QEH8_SORBI | F0F1-type ATP synthase, gamma subunit | 3.52 |
| tr\|A0A059Q225\|A0A059Q225_9POAL | F0F1-type ATP synthase, gamma subunit | 2.04 |
| tr\|A0A1B6QPK7\|A0A1B6QPK7_SORBI | Globins and related hemoproteins | -5.26 |
| tr\|A0A1D6KWT7\|A0A1D6KWT7_MAIZE | Glycerophosphoryl diester phosphodiesterase | -3.64 |
| tr\|A0A1Z5RBY4\|A0A1Z5RBY4_SORBI | Glycerophosphoryl diester phosphodiesterase | -2.13 |
| tr\|A0A1D6GAY6\|A0A1D6GAY6_MAIZE | Glycerophosphoryl diester phosphodiesterase | -1.58 |
| tr\|C5YYQ6\|C5YYQ6_SORBI | Inorganic pyrophosphatase/Nucleosome remodeling factor, subunit NURF38 | -2.18 |
| tr\|C5WYF2\|C5WYF2_SORBI | Malate dehydrogenase | -3.01 |
| sp\|P15719\|MDHP_MAIZE | Malate dehydrogenase | 1.96 |
| tr\|A0A3L6DPQ8\|A0A3L6DPQ8_MAIZE | MAM33, mitochondrial matrix glycoprotein | 2.34 |
| tr\|A0A1B6QHU0\|A0A1B6QHU0_SORBI | NAD-dependent malate dehydrogenase | 1.88 |
| tr\|A0A2Z5QFN6\|A0A2Z5QFN6_SACOF | NADH dehydrogenase subunits 2, 5, and related proteins | 1.68 |
| tr\|A0A3L6EB66\|A0A3L6EB66_MAIZE | NADH:ubiquinone oxidoreductase NDUFA2/B8 subunit | -2.37 |
| tr\|A0A317Y701\|A0A317Y701_MAIZE | NADH:ubiquinone oxidoreductase, NDUFB7/B18 subunit | 2.69 |
| tr\|A0A023Q225\|A0A023Q225_9POAL | NADH-cytochrome b-5 reductase | 2.05 |
| tr\|A0A1D6MK15\|A0A1D6MK15_MAIZE | NADH-cytochrome b-5 reductase | 2.15 |
| tr\|C5XND6\|C5XND6_SORBI | NADH-cytochrome b-5 reductase | 1.80 |
| tr\|C5Z889\|C5Z889_SORBI | NADH-dehydrogenase (ubiquinone) | 2.15 |
| tr\|C5WQR1\|C5WQR1_SORBI | NADH-ubiquinone oxidoreductase, subunit NDUFB10/PDSW | 1.95 |
| tr\|A0A1D6N0R1\|A0A1D6N0R1_MAIZE | NADP/FAD dependent oxidoreductase | 2.35 |
| tr\|C5YU98\|C5YU98_SORBI | NADP+-dependent malic enzyme | -4.31 |
| tr\|A0A2D0UJT5\|A0A2D0UJT5_9POAL | NADP-dependent isocitrate dehydrogenase | -2.92 |
| tr\|C5YNP6\|C5YNP6_SORBI | Predicted oxidoreductase | -2.16 |
| tr\|B8A299\|B8A299_MAIZE | Proteins containing the FAD binding domain | -3.67 |
| tr\|A0A1D6EVW0\|A0A1D6EVW0_MAIZE | Ubiquinol cytochrome c reductase assembly protein CBP3 | 3.54 |
| tr\|A0A3L6EX67\|A0A3L6EX67_MAIZE | Ubiquinol-cytochrome c reductase hinge protein | -2.13 |
| tr\|A0A1D6MAF6\|A0A1D6MAF6_MAIZE | UDP-glucuronosyl and UDP-glucosyl transferase | -2.16 |
| tr\|C5XHI6\|C5XHI6_SORBI | UDP-glucuronosyl and UDP-glucosyl transferase | -4.02 |
| tr\|C5YMV6\|C5YMV6_SORBI | UDP-glucuronosyl and UDP-glucosyl transferase | -3.26 |
| tr\|C5Z1F3\|C5Z1F3_SORBI | UDP-glucuronosyl and UDP-glucosyl transferase | -2.64 |
| tr\|A0A3L6F226\|A0A3L6F226_MAIZE | UDP-glucuronosyl and UDP-glucosyl transferase | -1.63 |
| tr\|A0A1D6LKC6\|A0A1D6LKC6_MAIZE | Voltage-gated shaker-like K+ channel, subunit beta/KCNAB | -2.15 |
| tr\|A0A1W0VYH4\|A0A1W0VYH4_SORBI | Voltage-gated shaker-like K+ channel, subunit beta/KCNAB | -3.14 |
| tr\|A0A317Y939\|A0A317Y939_MAIZE | Voltage-gated shaker-like K+ channel, subunit beta/KCNAB | -2.56 |
| tr\|A0A3L6F4Q6\|A0A3L6F4Q6_MAIZE | Voltage-gated shaker-like K+ channel, subunit beta/KCNAB | 2.25 |
| tr\|C5X1F2\|C5X1F2_SORBI | Voltage-gated shaker-like K+ channel, subunit beta/KCNAB | -4.00 |
| tr\|A0A1B6PHC9\|A0A1B6PHC9_SORBI | Zinc-binding oxidoreductase | 2.64 |
| tr\|A0A1Z5RQ67\|A0A1Z5RQ67_SORBI | Zinc-binding oxidoreductase | -3.13 |
| tr\|A0A3L6EP70\|A0A3L6EP70_MAIZE | Zinc-binding oxidoreductase | -2.60 |

Supplementary Table S2. Candidate DEPs associated with cell walls

| **Protein ID** | **Protein description** | **Difference multiple** |
| --- | --- | --- |
|  |  | **Treated1/ control** |
|  |  | **(Up/down 6/6)** |
| tr\|A0A096QRE7\|A0A096QRE7_MAIZE | Acetylglucosaminyl transferase EXT1/exostosin 1 | 2.08 |
| tr\|A0A1B6PNB8\|A0A1B6PNB8_SORBI | Apolipoprotein D/Lipocalin | 1.17 |
| tr\|A0A1B6Q537\|A0A1B6Q537_SORBI | Chitinase | -7.74 |
| tr\|A0A1D6FJ91\|A0A1D6FJ91_MAIZE | Acetylglucosaminyltransferase EXT2/exostosin 2 | 2.56 |
| tr\|A0A1D6NT62\|A0A1D6NT62_MAIZE | Glycosyltransferase | -2.34 |
| tr\|A0A317YA26\|A0A317YA26_MAIZE | UDP-glucose 4-epimerase/UDP-sulfoquinovose synthase | -3.53 |
| tr\|A0A3L6DNU2\|A0A3L6DNU2_MAIZE | Acetylglucosaminyltransferase EXT1/exostosin 1 | 2.37 |
| tr\|B4F9U8\|B4F9U8_MAIZE | dTDP-glucose 4-6-dehydratase/UDP-glucuronic acid decarboxylase | -2.38 |
| tr\|C5X399\|C5X399_SORBI | Pectin acetylesterase and similar proteins | 1.96 |
| tr\|C5XB38\|C5XB38_SORBI | Chitinase | -6.58 |
| tr\|C5Y5U9\|C5Y5U9_SORBI | Chitinase | -6.47 |
| tr\|Q38JD3\|Q38JD3_SACOF | Apolipoprotein D/Lipocalin | 2.19 |

Supplementary Table S3. Candidate DEPs associated with cell cycle

| **Protein ID** | **Protein description** | **Difference multiple** |
| --- | --- | --- |
|  |  | **Treated1/ control** |
|  |  | **(Up/down 5/7)** |
| tr\|A0A1D6IIL4\|A0A1D6IIL4_MAIZE | Anaphase promoting complex, Cdc20, Cdh1, and Ama1 subunits | -3.03 |
| tr\|A0A1D6FJH1\|A0A1D6FJH1_MAIZE | Apoptosis-related protein/predicted DNA-binding protein | -3.12 |
| tr\|A0A1D6K8R9\|A0A1D6K8R9_MAIZE | Casein kinase II, beta subunit | 2.63 |
| tr\|A0A059PYU0\|A0A059PYU0_9POAL | Cell cycle-associated protein Mob1-1 | -2.21 |
| tr\|A0A194YN98\|A0A194YN98_SORBI | Centromere/kinetochore protein zw10 involved in mitotic chromosome segregation | -2.15 |
| tr\|C5YCJ4\|C5YCJ4_SORBI | Checkpoint 9-1-1 complex, HUS1 component | 2.47 |
| tr\|A0A1D6NWY2\|A0A1D6NWY2_MAIZE | Defender against cell death protein/oligosaccharyltransferase, epsilon subunit | 1.64 |
| tr\|A0A1D6LRY4\|A0A1D6LRY4_MAIZE | Microtubule-associated protein essential for anaphase spindle elongation, MAP65-1a | -2.00 |
| tr\|C5XN19\|C5XN19_SORBI | MORC family ATPases | 2.42 |
| tr\|A0A096SC75\|A0A096SC75_MAIZE | Protein kinase ATM/Tel1, involved in telomere length regulation and DNA repair | -1.76 |
| tr\|C5X7T2\|C5X7T2_SORBI | Protein predicted to be involved in spindle matrix formation, contains DM13, DoH, and DOMON domains | 2.06 |
| tr\|A0A1B6Q961\|A0A1B6Q961_SORBI | Speckle-type POZ protein SPOP and related proteins with TRAF, MATH and BTB/POZ domains | -2.22 |

Supplementary Table S4. Candidate DEPs associated with synthesis of secondary metabolites

| **Category** | **Protein ID** | **Protein description** | **Difference multiple** |
| --- | --- | --- | --- |
|  |  |  | **treated1/ control** |
|  |  |  | **(up/down 1/6)** |
| Biosynthesis of scopolamine, pethidine and pyridine alkaloids | tr\|A0A194YNT9\|A0A194YNT9_SORBI | Aspartate aminotransferase/Glutamic oxaloacetic transaminase AAT1/GOT2 | -1.49 |
|  | tr\|A0A3L6EK06\|A0A3L6EK06_MAIZE | Aspartate aminotransferase/Glutamic oxaloacetic transaminase AAT1/GOT2 | -1.14 |
| Biosynthesis of styrene acrylic | tr\|A0A160EBB1\|A0A160EBB1_9POAL | Cytochrome P450 CYP2 subfamily | -5.43 |
|  | tr\|A0A1W0VRT6\|A0A1W0VRT6_SORBI | Alcohol dehydrogenase, class V | -2.17 |
|  | tr\|A0A317Y9B9\|A0A317Y9B9_MAIZE | Protein ECERIFERUM 26-like | 1.77 |
|  | tr\|A0A3L6EYC8\|A0A3L6EYC8_MAIZE | Agmatine coumaroyltransferase-2 | -1.59 |
|  | tr\|C5XNX7\|C5XNX7_SORBI | Flavonol reductase/cinnamoyl-CoA reductase | -2.68 |

Supplementary Table S5.Candidate DEPs associated with antioxidant

| **Protein ID** | **Protein description** | **Difference multiple** |
| --- | --- | --- |
|  |  | **Treated1/ control** |
|  |  | **(Up/down 13/41)** |
| tr\|A0A317YJY0\|A0A317YJY0_MAIZE | - | -1.20 |
| tr\|C5WZG9\|C5WZG9_SORBI | 3-oxoacyl CoA thiolase | 1.63 |
| tr\|A0A3L6FAH5\|A0A3L6FAH5_MAIZE | Alkyl hydroperoxide reductase/peroxiredoxin | -1.08 |
| tr\|C5XG44\|C5XG44_SORBI | Alkyl hydroperoxide reductase/peroxiredoxin | -3.41 |
| tr\|S4WDN5\|S4WDN5_9POAL | Ascorbate peroxidase | -2.77 |
| tr\|A0A075TDY4\|A0A075TDY4_9POAL | Ascorbate peroxidase | -1.00 |
| tr\|A0A1B6QQQ9\|A0A1B6QQQ9_SORBI | Catalase | -2.56 |
| tr\|A0A059Q0Q4\|A0A059Q0Q4_9POAL | Catalase | -1.75 |
| tr\|A0A1B6P6Z1\|A0A1B6P6Z1_SORBI | Glutaryl-CoA dehydrogenase | 2.60 |
| tr\|A0A059Q2U8\|A0A059Q2U8_9POAL | Glutathione peroxidase | -3.00 |
| tr\|A0A194YS45\|A0A194YS45_SORBI | Glutathione peroxidase | -2.05 |
| tr\|A0A059Q2U8\|A0A059Q2U8_9POAL | Glutathione peroxidase | -3.00 |
| tr\|A0A0K0QRE9\|A0A0K0QRE9_SACOF | Glutathione S-transferase | -3.02 |
| tr\|A0A1B6Q818\|A0A1B6Q818_SORBI | Glutathione S-transferase | -1.25 |
| tr\|A0A1B6QNZ1\|A0A1B6QNZ1_SORBI | Glutathione S-transferase | -2.35 |
| tr\|A0A1D6PD99\|A0A1D6PD99_MAIZE | Glutathione S-transferase | -2.89 |
| tr\|A0A1D6QQD4\|A0A1D6QQD4_MAIZE | Glutathione S-transferase | -2.80 |
| tr\|A0A317Y1Z9\|A0A317Y1Z9_MAIZE | Glutathione S-transferase | -2.79 |
| tr\|A0A3L6E750\|A0A3L6E750_MAIZE | Glutathione S-transferase | -1.85 |
| tr\|C5XKV8\|C5XKV8_SORBI | Glutathione S-transferase | -2.61 |
| tr\|A0A317Y5S8\|A0A317Y5S8_MAIZE | Glutathione S-transferase | -2.01 |
| tr\|A0A3L6DAR9\|A0A3L6DAR9_MAIZE | Glutathione S-transferase | 3.02 |
| tr\|A0A1B6PCZ5\|A0A1B6PCZ5_SORBI | Glutathione S-transferase | -2.11 |
| tr\|A0A317YFV6\|A0A317YFV6_MAIZE | L-ascorbate peroxidase 1 | -3.23 |
| tr\|A0A1B6P680\|A0A1B6P680_SORBI | Long-chain acyl-CoA synthetases (AMP-forming) | 3.43 |
| tr\|A0A1B6PSR5\|A0A1B6PSR5_SORBI | Long-chain acyl-CoA synthetases (AMP-forming) | 2.97 |
| tr\|B7ZX64\|B7ZX64_MAIZE | Long-chain acyl-CoA synthetases (AMP-forming) | 3.60 |
| tr\|A0A2D0UJT5\|A0A2D0UJT5_9POAL | NADP-dependent isocitrate dehydrogenase | -2.92 |
| tr\|A0A0A7DVW9\|A0A0A7DVW9_9POAL | Peroxidase | -4.24 |
| tr\|A0A1B6PLE8\|A0A1B6PLE8_SORBI | Peroxidase | 3.22 |
| tr\|A0A1D6FUY8\|A0A1D6FUY8_MAIZE | Peroxidase | -5.50 |
| tr\|A0A1D6N0K1\|A0A1D6N0K1_MAIZE | Peroxidase | -7.54 |
| tr\|A0A1Z5RMP0\|A0A1Z5RMP0_SORBI | Peroxidase | -7.32 |
| tr\|A0A3L6GBT3\|A0A3L6GBT3_MAIZE | Peroxidase | -4.86 |
| tr\|C5WRN5\|C5WRN5_SORBI | Peroxidase | 2.60 |
| tr\|C5WVK2\|C5WVK2_SORBI | Peroxidase | -5.57 |
| tr\|C5XIN9\|C5XIN9_SORBI | Peroxidase | -7.33 |
| tr\|C5YQ75\|C5YQ75_SORBI | Peroxidase | -7.49 |
| tr\|C5YZI4\|C5YZI4_SORBI | Peroxidase | -6.18 |
| tr\|C5Z469\|C5Z469_SORBI | Peroxidase | -8.61 |
| tr\|C5Z475\|C5Z475_SORBI | Peroxidase | -4.68 |
| tr\|C5Z8S7\|C5Z8S7_SORBI | Peroxidase | -4.81 |
| tr\|B6SIU4\|B6SIU4_MAIZE | Peroxidase | -1.07 |
| tr\|C5X746\|C5X746_SORBI | Peroxidase | 1.21 |
| tr\|B6T3V1\|B6T3V1_MAIZE | Peroxidase | -3.10 |
| tr\|A0A1W0VX32\|A0A1W0VX32_SORBI | Peroxidase | -1.35 |
| tr\|A0A3L6G3E7\|A0A3L6G3E7_MAIZE | Peroxidase | 1.98 |
| sp\|A5H452\|PER70_MAIZE | Peroxidase 70 | -5.07 |
| tr\|A0A1B6PMT8\|A0A1B6PMT8_SORBI | PEROXIDASE_4 | 2.41 |
| tr\|A0A1Z5RCK3\|A0A1Z5RCK3_SORBI | PEROXIDASE_4 domain-containing protein | 1.28 |
| tr\|A0A1D6MDL7\|A0A1D6MDL7_MAIZE | Peroxisomal membrane anchor protein (peroxin) | -1.94 |
| tr\|A0A1D6H6N7\|A0A1D6H6N7_MAIZE | Peroxisomal membrane protein MPV17 and related proteins | 1.55 |
| tr\|C5WNQ9\|C5WNQ9_SORBI | Peroxisomal phytanoyl-CoA hydroxylase | -1.81 |
| tr\|A0A3L6E4D3\|A0A3L6E4D3_MAIZE | Possible oxidoreductase | -2.20 |
