# Supplementary material for: An integrated physiology, cytology, and proteomics analysis reveals a network of sugarcane protoplast responses to enzymolysis
Source: Front Plant Sci. 2022 Nov 28;13:1066073. doi: 10.3389/fpls.2022.1066073 (PMC9744229; doi:10.3389/fpls.2022.1066073)
Supplement: Supplementary file 2 [file DataSheet_2.docx]

**Supplementary Table S6.** Primers for qRT-PCR.

| **Gene** | **Primer 5′－3′** | |
| --- | --- | --- |
| Cu/Zn SOD | F | GGTTCGATGCTGAGTTGAGTC |
|  | R | CTGCGTAAGTGATACCTGGGT |
| CAT | F | CCACCACAACAACCACTACG |
|  | R | GCAATCACAGTCTTGTCTCGG |
| DREB | F | GCCTGGTACATCATGCGAGT |
|  | R | CCATTCTGCCTTTAGGGAGC |
| WRKY | F | GAGAACTTCATGGCCGTCAAG |
|  | R | TTCAGCTCCGTGTACTTGGC |
| MAPK4 | F | CACAAGTACCAGCCTCCCAT |
|  | R | CCATCTCCCTCGTCTCAAAGT |
| NAC | F | TGGTGATGTACTACCTCCTCCG |
|  | R | GAAGAAGTACCACTCCTTCTCCC |
| Cyclin D3 | F | GGAGGATGCAAGCTGTGACT |
|  | R | GCCACCCTCTCAAACATGGA |
| Cyclin A | F | CAAGGACA AGGAGCAGAGCAATGG |
|  | R | CCTTCACATCTGGCTGCTGCTG |
| Cyclin B | F | GGAGTTGGTCGCTAGTGTATCTGC |
|  | R | ACCGAGTGACCGACAGAGATGAG |
| Cdc 2 | F  R | GCAGCACAGCAACATTGTCA  CTTCAGTGAGTTTGTGCGGC |
| PSK | F  R | CGACGACGAGGAGGAAGAGGAG  CAGGCGTTCTCGGTGTAGTTGC |
| CESA | F  R | GACTGGTGGAGAAACGAGCA  TTGCCTTCGAGGTAACCGTG |
| GAUT | F | GCCACGGCTTCCATGTCAATCC |
|  | R | CGAGGCTGGCGATCAATCTACTG |
| GADPH | F | AAGGGTGGTGCCAAGAAGG |
|  | R | CAAGGGGAGCAAGGCAGTT |
