# Supplementary material for: An integrated physiology, cytology, and proteomics analysis reveals a network of sugarcane protoplast responses to enzymolysis
Source: Front Plant Sci. 2022 Nov 28;13:1066073. doi: 10.3389/fpls.2022.1066073 (PMC9744229; doi:10.3389/fpls.2022.1066073)
Supplement: Supplementary file 3 [file DataSheet_3.doc]

**The raw data of Figure 2, Figure 3 and Figure 4.**

| Figure 2 |  |  | sample1 | sample2 | sample3 | Average value | variance |
| --- | --- | --- | --- | --- | --- | --- | --- |
|  | MDA | young leaves | 1.1 | 1.3 | 1.3 | 1.23333 | 0.11547 |
|  |  | protoplast | 5.33 | 5.66 | 5.74 | 5.57667 | 0.21733 |
|  |  |  |  |  |  |  |  |
|  | O2- | young leaves | 35 | 35.4 | 34.9 | 35.1 | 0.26458 |
|  |  | protoplast | 0.35 | 0.48 | 0.47 | 0.43333 | 0.07234 |
|  |  |  |  |  |  |  |  |
|  | SOD | Young leaves | 1720 | 1509 | 1480 | 1569.66667 | 130.99746 |
|  |  | Protoplast | 1398 | 1433 | 1476 | 1435.66667 | 39.06832 |
|  |  |  |  |  |  |  |  |
|  | POD | Young leaves | 9.8 | 9.5 | 8.7 | 9.33333 | 0.56862 |
|  |  | Protoplast | 1.79 | 1.6 | 1.56 | 1.65 | 0.12288 |
|  |  |  |  |  |  |  |  |
|  | CAT | Young leaves | 84.7 | 87.2 | 86.1 | 86 | 1.253 |
|  |  | Protoplast | 5.37 | 5.75 | 5.73 | 5.61667 | 0.21385 |
|  |  |  |  |  |  |  |  |
|  | APX | Young leaves | 8.2 | 9.3 | 9.1 | 8.86667 | 0.58595 |
|  |  | Protoplast | 1.6 | 1.68 | 1.37 | 1.55 | 0.16093 |
|  |  |  |  |  |  |  |  |
| Figure 3 | Cu/ZnSOD | Young leaves | 35 | 35.7 | 35.4 | 35.36667 | 0.35119 |
|  |  | Protoplast | 0.5 | 0.61 | 0.57 | 0.56 | 0.05568 |
|  |  |  |  |  |  |  |  |
|  | CAT | Young leaves | 28.81 | 29.79 | 29.5 | 29.36667 | 0.50342 |
|  |  | Protoplast | 0.7 | 0.89 | 0.87 | 0.82 | 0.1044 |
|  |  |  |  |  |  |  |  |
| Figure 4 | DREB | Young leaves | 1.48 | 1.64 | 1.53 | 1.55 | 0.08185 |
|  |  | Protoplast | 19 | 21.9 | 23 | 21.3 | 2.0664 |
|  |  |  |  |  |  | -- | -- |
|  | WRKY | Young leaves | 280 | 354 | 393 | 342.33333 | 57.39628 |
|  |  | Protoplast | 59176 | 58420 | 58513 | 58703 | 412.26084 |
|  |  |  |  |  |  | -- | -- |
|  | MAPK4 | Young leaves | 109 | 130 | 103 | 114 | 14.17745 |
|  |  | Protoplast | 6603 | 6780 | 7074 | 6819 | 237.90965 |
|  |  |  |  |  |  | -- | -- |
|  | NAC | Young leaves | 96 | 114 | 101 | 103.66667 | 9.29157 |
|  |  | Protoplast | 207570 | 209300 | 208668 | 208512.6667 | 875.39781 |

Please refer to the paper chart for detailed units of data.
